# Antibiotic resistance of uropathogens among the community-dwelling pregnant and nonpregnant female: a step towards antibiotic stewardship

**DOI:** 10.1186/s12879-022-07914-1

**Published:** 2022-12-13

**Authors:** Sarita Mohapatra, Shwetha J. Venugopal, Mani Kalaivani, Shashi Kant, Vibhor Tak, Rajashree Panigrahy, Sneha K. Chunchanur, Deepak Kocher, Birasen Behera, Swati Pundir, Susmita Chaudhuri, Hitender Gautam, Seema Sood, Bimal Kumar Das, Arti Kapil, Arvind Kumar, Rajesh Kumari, R. Ambica, Pankaj Hari, Sumit Malhotra, Harsal Ramesh Salve, CAUTION-ED Study (Community-acquired UTI, Emerging Drug Resistance), Sarita Mohapatra, Shwetha J. Venugopal, Vibhor Tak, Rajashree Panigrahy, Sneha KChunchanur, Susmita Chaudhuri, Pankaj Hari, Suren Das, Pankaja Ravi Raghav, Shashi Kant

**Affiliations:** 1 Department of Microbiology, All India Institute of Medical Sciences, New Delhi, India; 2 Department of Microbiology, Bangalore Medical College and Research Institute, Bangalore, India; 3 Department of Biostatistics, All India Institute of Medical Sciences, New Delhi, India; 4 Centre for Community Medicine, All India Institute of Medical Sciences, New Delhi, India; 5 Department of Microbiology, All India Institute of Medical Sciences, Jodhpur, India; 6 Department of Microbiology, Institute of Medical Sciences and Sum Hospital, Bhubaneswar, India; 7 Translational Health Science and Technology Institute, Faridabad, India; 8 Department of Medicine, All India Institute of Medical Sciences, New Delhi, India; 9 Department of Obstetrics and Gynaecology, All India Institute of Medical Sciences, New Delhi, India; 10 Department of Pediatrics, All India Institute of Medical Sciences, New Delhi, India

**Keywords:** Anti-microbial resistance, Pregnant women, Urinary tract infection, Uropathogen

## Abstract

**Background:**

Indiscriminate and widespread use of antibiotics has resulted in emergence of many antibiotic-resistant organisms. Antibiotic administration during pregnancy is mostly avoided, unless there is compelling medical condition. We hypothesized that the uropathogens isolated from pregnant women would be more susceptible to antibiotics compared to those isolated from nonpregnant women, thus will be helpful in formulating separate empiric guideline for pregnant women based on the resistance pattern.

**Methods:**

This was a prospective cross-sectional study conducted over a period of 2 years in which females with the clinical diagnosis of either cystitis or asymptomatic bacteriuria during pregnancy were included from the community settings. Uropathogen species and their antimicrobial resistance pattern were compared between the pregnant and nonpregnant groups. After accounting for centre-to-centre variation and adjusting for age and socio-economic status, the adjusted odds ratio for antibiotic resistance was calculated and compared between pregnant and nonpregnant women using logistic regression analysis.

**Results:**

A total of 1758 women (pregnant: 43.3%; nonpregnant: 56.6%) were screened in the study over a period of 2 years, out of which 9.3% (163/1758) were having significant bacteriuria. *Escherichia coli* and *Klebsiella pneumoniae* were the two commonest uropathogen in both the groups; their prevalence being 83.6% in pregnant women and 85.2% in nonpregnant women, respectively. Resistance against ampicillin, cefixime, cefoxitin, ceftazidime, ceftriaxone and amoxicillin-clavulanic acid were found significantly lower in the pregnant women compared to nonpregnant. After adjusting the age and socio-economic status accounting for centre-to-centre variation, the odds of resistance for cefixime, amoxicillin-clavulanic acid and co-trimoxazole were found lower and statistically significant among the pregnant women group.

**Conclusions:**

The antimicrobial resistance was significantly higher among the community-dwelling nonpregnant women compared to pregnant women in case of few antibiotics. The study highlighted the need of building local antibiogram that could help to initiate the empirical treatment and thus prevent emergence of antimicrobial resistance.

## Background

Anti-microbial resistance (AMR) is a serious global health problem. Urine is the commonest specimen sent to the microbiology department for culture and antibiotic susceptibility test (AST) [1]. Also, clinical suspicion of urinary tract infection (UTI) without ordering urine culture has been reported as one of the major drivers for an irrational antibiotic prescription [1]. Inappropriate usage of antibiotics resulted in the development of resistant microorganisms worldwide [2]. Antibiotic usage during pregnancy is the least unless there is a compelling medical condition such as UTI, sexually transmitted infections, or respiratory tract infections. Hence, we hypothesized that the uropathogens isolated from pregnant women would be more susceptible to antibiotics compared to those isolated from nonpregnant women, who may have been exposed to antibiotics in the recent past. Therefore, we aimed to compare the prevalence of uropathogen and the AMR pattern among pregnant and nonpregnant women. This study data might serve as supporting evidence in formulation of separate empiric guideline for pregnant women based on the resistance pattern.

## Methods

This study was a part of an ongoing multi-centric study on community-acquired UTI and emerging drug resistance (CAUTION-ED study group, grant funded by ICMR, 2019). Ethical approval had been obtained from the institute ethical committee (IEC-192/05.04.2019, RP-28/2019). Consecutive women presenting to the outpatient department of the community health center at four different geographical sites of India with clinical diagnosis of UTI were included in this study. Pregnant females were included in this study if microbiologically detected to have asymptomatic bacteriuria (ASB) during the antenatal check-up. The study period was conducted over a period of 2 years (Oct, 2019–Oct, 2021).

Consecutive patients with age above 16 years and less than 50 years from the defined community presenting to the outpatient department with a diagnosis of uncomplicated UTI (without any anatomical or functional abnormality) showing symptoms of cystitis, burning micturition, increase in frequency, urgency while passing urine, pain above pubic symphysis, asymptomatic bacteriuria in pregnant women, history of recurrent episodes of UTI (at least 2 episodes within 6 months or 3 episodes in a year) and fever without focus were included in this study. Patients with asymptomatic bacteriuria in conditions other than pregnancy, history of hospitalization 1 week prior to presentation, history of hospitalization for more than 48 h, known case of neurogenic bladder, obstructive uropathy, pediatric patients with vesicoureteral reflux and on antibiotic prophylaxis were excluded from the study.

Clean catch mid-stream urine sample was collected from the patients and transported using 1.8% boric acid transport vial to the referral tertiary care health centers for further processing. Urine sample was collected, and processed on the Cystine Lactose Electrolyte Deficient Agar (CLED) as per standard operative protocol. Monomicrobial uropathogen as pure culture with significant bacteriuria (**≥ **10^3^ CFU/ml for acute cystitis and ≥ 10^5^ CFU/ml for asymptomatic bacteriuria among the pregnant females) were further tested for their identification using Matrix Assisted Laser Desorption Ionization Time of Flight Mass Spectrometry (MALDI-TOF MS) (VITEK-MS system, BioMérieux, Marcy-l’Étoile, France). Antimicrobial susceptibility testing (AST) was performed using VITEK-2 (Biomeriux, Durhum, US) system. Clinical & Laboratory Standards Institute M100 was followed as the reference guideline for antibiotics breakpoints. Uropathogens along with their AMR pattern were compared between the pregnant and nonpregnant women group.

Statistical analysis was carried out using Stata 16.0 (Stata Corp. LLC). Data was summarized as mean ± standard deviation and number (%). Demographic characteristics such as age, education status and socio-economic status (SES) were compared between pregnant and nonpregnant women using t-test (continuous variables) and chi-square test (categorized variables) [3, 4]. The AMR pattern of uropathogens between pregnant and nonpregnant women was compared using z-test for proportions. Multilevel mixed effect model for binary responses with center as random effect was used to compare the resistance against various antibiotics among pregnant and non-pregnant women after adjusting for variability due to age and SES parameters. Both unadjusted and adjusted odds ratio along with 95% confidence interval were reported. A *p*-value of less than 0.05 was considered statistically significant.

## Results

**Fig. 1:**
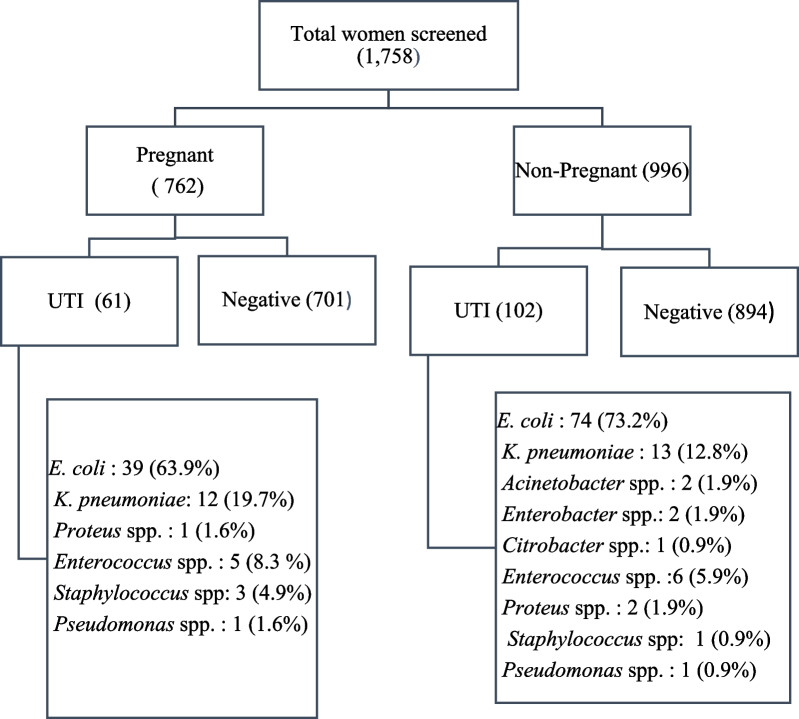
Flow chart showing breakup of participants in the study

Total 1758 female patients were screened in the study over a period of 2 years [pregnant: 762, (43.3%); nonpregnant: 996, (56.6%)]. Approximately, 9.3% of the total patient (163/1758) were having significant bacteriuria [pregnant: 61, (37.4%); nonpregnant: 102, (62.6%)] (Fig. 1).

The demographic distribution among pregnant vs nonpregnant women with significant bacteriuria has been presented in Table 1. The majority of patients belong to the young age group 19-35 years (122, 74.8%) in both groups, followed by others [16–18 years (3, 1.8%); 36–50 years (38, 23.3%)]. More than 60% of the total patients belong to the poor and very poor class (100, 61.3%) in comparison to the middle class (52, 32%) and the rich class (11, 6.7%). The maximum level of education among most of the females observed to be secondary (50, 30.6%) in both the groups followed by higher (49, 30%); primary (33, 20.2%); and not known (31, 19%).

**Table 1:** Demographic distribution among pregnant and nonpregnant females with significant bacteriuria

Demographic Profile	Pregnant n = 61 (8%)	Nonpregnant n = 102 (10.2%)	Total n = 163 (10.7%)
Age (in years)			
16–18	2 (3.2%)	1 (0.9%)	3 (1.8%)
19–35	57 (93.4%)	65 (63.7%)	122 (74.8%)
36–50	2 (3.2%)	36 (35.2%)	38 (23.3%)
Education			
Primary	16 (26.2%)	17 (16.6%)	33 (20.2%)
Secondary	25 (41%)	25 (24.5%)	50 (30.6%)
Higher	20 (32.7%)	29 (28.4%)	49 (30%)
Not known	0	31 (30.3%)	31 (19%)
Socio-economic status			
Poor and very poor	53 (86.8%)	47 (46%)	100 (61.3%)
Middle class	7 (11.4%)	45 (44%)	52 (32%)
Rich	1 (1.6%)	10 (9.8%)	11 (6.7%)

*Escherichia coli* and *Klebsiella pneumoniae* were observed to be the commonest uropathogens in both pregnant and nonpregnant group (pregnant: 83.6%, nonpregnant: 85.2%). The other less frequent uropathogens detected among the GNBs (Gram-Negative bacilli) were *Proteus* spp., *Pseudomonas* spp., *Acinetobacter* spp., and *Enterobacter* spp.. While *Staphylococcus* spp., *Enterococcus* spp. and *Streptococcus agalactiae* were detected among the Gram-Positive cocci. AMR profiles of the major uropathogens *E. coli* and *Klebsiella pneumoniae* were compared among both groups (Table 2). The susceptibility was observed higher for most antibiotics in the pregnant group in comparison to nonpregnant group except for ciprofloxacin, piperacillin-tazobactam, gentamicin, nitrofurantoin and ertapenem. Fosfomycin, which is only tested in *E. coli* isolates showed 100% susceptibility in both groups. On analysis of the unadjusted odds ratio, significant difference in the resistance pattern for *E. coli* and *K. pneumoniae* was found between pregnant and nonpregnant women groups for ampicillin, cefixime, cefoxitin, ceftazidime, ceftriaxone and amoxicillin-clavulanic acid (*p* < 0.05) (Table 2). After accounting for centre-to-centre variation and adjusting for variables age and SES in the analysis of the adjusted odds ratio, only cefixime and amoxicillin-clavulanic acid remained statistically significant. Co-trimoxazole was found statistically significant in the adjusted analysis.

**Table 2:** Antimicrobial resistance pattern of predominant gram-negative bacilli (*E. coli* and *K. pneumoniae* only) among pregnant and nonpregnant females

Antibiotics	Pregnant GNB* n = 51 (6.2%)	Nonpregnant GNB* n = 86 (8.7%)	*p-*value	Odd ratio (95% confidence interval)
Ampicillin	31 (60.8%)	60 (69.8%)	**0.03	2.49 (1.09, 5.66)
Ticarcillin	31(60.8%)	56 (65.1%)	0.06	2.15 (0.97, 4.76)
Cefixime	18 (35.3%)	47 (54.7%)	**0.01	3.34 (1.57, 7.11)
Cefoxitin	8 (15.7%)	27 (31.4%)	**0.02	3.21 (1.18, 8.74)
Ceftazidime	12 (23.5%)	35 (40.7%)	**0.02	2.81 (1.21, 6.53)
Ceftriaxone	15 (29.4%)	44 (51.2%)	**0.01	2.86 (1.26, 6.51)
Co-trimoxazole	17 (33.3%)	41 (47.7%)	0.07	2.95 (0.91, 9.63)
Ciprofloxacin	28 (54.9%)	46 (53.5%)	0.52	1.38 (0.52, 3.65)
Ofloxacin	18 (35.3%)	32 (37.2%)	0.84	0.9 (0.31, 2.61)
Gentamicin	8 (15.7%)	12 (14.0%)	0.92	0.95 (0.32, 2.83)
Amikacin	1 (2.0%)	7 (8.1%)	0.15	4.73 (0.56, 39.63)
Amoxicillin-clavulanic acid	16 (31.4%)	38 (44.2%)	**0.01	4.65 (1.39, 15.58)
Piperacillin-Tazobactam	15 (29.4%)	23 (26.7%)	0.76	1.18 (0.41, 3.43)
^#^Fosfomycin	0 (0.0%)	0 (0.0%)	–	–
Nitrofurantoin	15 (29.4%)	23 (26.7%)	0.97	0.98 (0.45, 2.14)
Ertapenem	3 (5.9%)	2 (2.3%)	0.38	0.44 (0.07, 2.76)

^*^GNB (here GNB includes the data of the predominant uropathogens i.e. *E.coli* and *K. pneumoniae* only)

^#^Fosfomycin was tested against *E. coli* isolates only

Table illustrates the resistance against the antibiotic between nonpregnant and pregnant women and **depicts statistically significant result (*p-*value < 0.05)

**Fig. 2:**
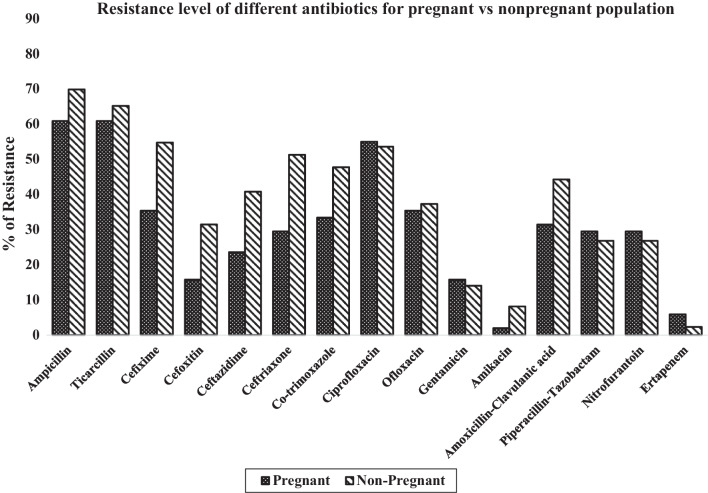
Bar diagram showing antimicrobial resistance pattern against antibiotics used for UTI treatment among pregnant and nonpregnant females

The AMR rate against the antibiotics used for GNB treatment between the pregnant and nonpregnant women is shown in Fig. 2.

**Fig. 3:**
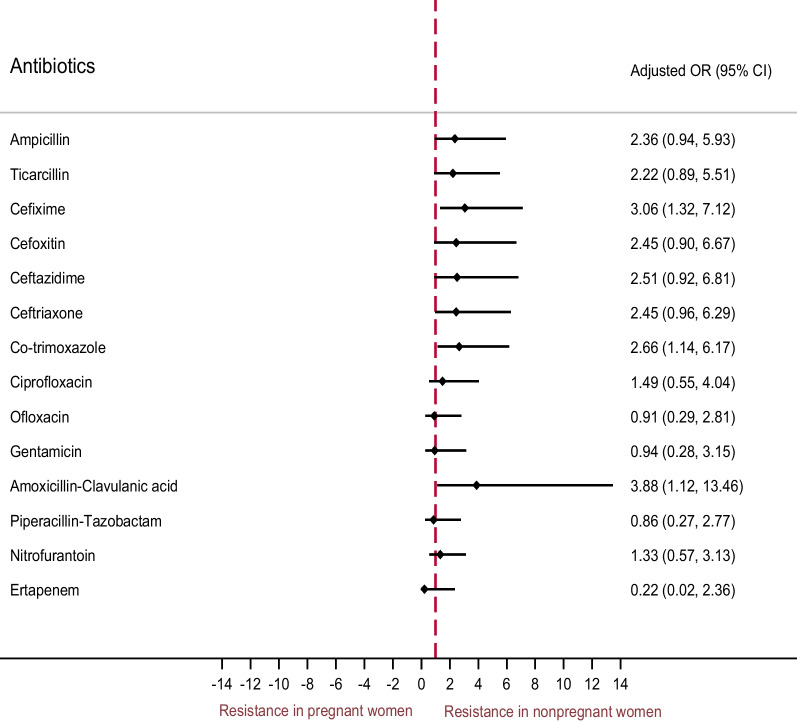
Forest plot showing adjusted analysis used for various antibiotic resistance between nonpregnant and pregnant females

The adjusted odds for AMR was higher in nonpregnant women compared to pregnant women for few of the antibiotics as represented in the forest plot (Fig. 3). Amikacin was excluded from the forest plot because the 95% confidence interval was too wide for any meaningful interpretation.

## Discussion

Increased usage of the antibiotics over the years has contributed to the emergence of more drug resistance in bacteria. [5, 6] The usage of antibiotics has been high particularly for UTI [7]. In addition, most of the UTIs were treated with empirical antibiotics without urine culture and AST [8]. Such situation is likely to favor emergence of resistant species.

Our study result highlights a significant difference in the antibiotic resistance data against many antibiotics in both pregnant and nonpregnant groups. More than 50% of the isolates were resistant to the common first line antibiotics such as ampicillin, ticarcillin and ciprofloxacin. The percentage of resistance against co-trimoxazole and beta-lactam/beta-lactamase inhibitors (BL/BLIs) ranged between 25 and 50%. Oral and intravenous cephalosporins such as cefixime, cefoxitin, ceftazidime and ceftriaxone along with ofloxacin showed resistance levels between 15 and 55%. While for amikacin, gentamicin and ertapenem resistance levels were less than 16%. Studies from Iran showed a similar pattern of resistance against first-line antibiotics. [9] In fact, the resistance against aminoglycoside, BL/BLIs in our study was much higher in the nonpregnant group. Hence, empirical treatment by commonly used first-line antibiotics is not only likely to fail but also contribute to the further emergence of AMR. Based on our results of resistance pattern in the community setting, we suggest nitrofurantoin (not in case of glucose-6-phosphate dehydrogenase deficiency, complicated UTI and in 3rd Trimester of pregnancy), followed by oral cephalosporin cefixime is the second choice for empirical treatment of UTI in pregnant females. If needed amoxicillin-clavulanic acid (strictly as per this study results only) or intravenous drugs such as fluoroquinolones/aminoglycosides/carbapenem/other beta-lactams/beta-lactamase inhibitors based on local antibiogram and culture AST may be used as an alternate therapy for the treatment. For nonpregnant females, we again suggest nitrofurantoin for the empirical treatment of UTI. Keeping in mind the high resistance rates observed and taking a conservative approach, we suggest sicker patients may be treated under inpatient care with optimal drug usage. Based on this study, we suggest the use of aminoglycosides (gentamicin or amikacin) or IV cephalosporins (i.e. cefoxitin) or piperacillin-tazobactam, and in case clinical setting demands BL/BLI combination/ carbapenem may also be considered as a salvage therapy and further modifying it on culture report. Fosfomycin has shown excellent susceptibility in treating *E. coli* infections, and is also tolerated well in both pregnant and nonpregnant females and should be kept as reserve choice to avoid its abuse in the community setting. In the present study, no resistance against fosfomycin was observed. Similar studies could be conducted in different geographical areas to generate their local antibiograms in the community settings. Thus, an initial empirical treatment based on the evidence generated could be started that can be modified whenever the urine culture and AST become available. This strategy could help to prevent the emergence of antibiotic-resistant organism. *E. coli* remained the commonest uropathogen in both pregnant and non-pregnant UTI cases. Although there are definite recommendations to treat ASB (i.e. pregnancy and before invasive urologic procedures), intake of antibiotic has been observed in many of ASB conditions. [10, 11] Antimicrobial stewardship programs at the community settings would be helpful to optimize the correct treatment of infection and thereby; reduce the emergence AMR.

The study has few limitations. The study was conducted at community settings catering rural populations in which the majority belong to the poor class without higher education. Hence, the exact data about antibiotic consumption in the recent past could not be obtained from the patients. Future studies can be planned with exact antibiotic consumption data, that can be further correlated with the antibiotic resistance in both the groups.

## Conclusions

The prevalence of AMR among women (both pregnant and nonpregnant) in the community was high. Resistance to antibiotics was generally higher among nonpregnant women compared to pregnant women. It highlights the need for urine culture, AST and routine surveillance to build the local antibiogram for the appropriate empirical therapy at the community settings.

## Data Availability

The data supporting the conclusions of this article are included within this published article only.

## Abbreviations

AMR: Antimicrobial resistance
AST: Antibiotic susceptibility test
UTI: Urinary tract infection
ASB: Asymptomatic bacteriuria
CLED: Cystine lactose electrolyte deficient agar
MALDI-TOF MS: Matrix assisted laser desorption ionization time of flight mass spectrometry
SES: Socio-economic status
GNB: Gram-negative bacilli
BL/BLI: Beta-lactam/beta-lactamase inhibitors
